# Validation of new Japanese classification system for esophageal achalasia

**DOI:** 10.1007/s10388-019-00658-z

**Published:** 2019-02-15

**Authors:** Ryo Kato, Kiyokazu Nakajima, Tsuyoshi Takahashi, Koji Tanaka, Yasuhiro Miyazaki, Tomoki Makino, Yukinori Kurokawa, Makoto Yamasaki, Masaki Mori, Yuichiro Doki

**Affiliations:** 1 Department of Gastroenterological Surgery, Osaka University, Graduate School of Medicine, 2-2, E-2, Yamadaoka, Suita, Osaka 565-0871 Japan; 2 Division of Next Generation Endoscopic Intervention (Project ENGINE), Global Center for Medical Engineering and Informatics, Osaka University, Center of Medical Innovation and Translational Research, 2-2, Yamadaoka, Suita, Osaka 565-0871 Japan

**Keywords:** Achalasia, Heller–Dor surgery, New Japanese classification system

## Abstract

**Background:**

The fourth edition of New Japanese classification system for esophageal achalasia was revised after a long interval of 30 years in 2012. In this new system, achalasia is morphologically classified into 3 types, based on its X-ray findings. However, the system has been limitedly used in Japan and has not been fully validated in terms of its predictive capability of postoperative outcomes. The purpose of this study was to clarify the validity of new Japanese classification system for esophageal achalasia, as an index of patient characteristics and as a predictor of operative and mid/long-term postoperative outcomes.

**Patients and methods:**

Fifty-nine cases of achalasia underwent laparoscopic Heller–Dor surgery between 2005 and 2018. We evaluated retrospectively patient characteristics, intraoperative findings, esophageal manometry, 24-h pH monitoring and postoperative course.

**Results:**

There were 34 St and 25 Sg/aSg cases. Age of St group was lower than Sg group. Preoperative duration of disease of St group was shorter than Sg. There were no differences in the results of surgical outcomes and prognoses.

**Conclusion:**

The new Japanese classification system may give additional insight and information in understanding epidemiology of esophageal achalasia; however, our study failed to demonstrate “inter-disease type” differences in surgical outcomes and prognoses.

## Introduction

Esophageal achalasia is an esophageal motility disorder of unknown etiology that results in impaired relaxation of the lower esophageal sphincter (LES) and loss of esophageal peristalsis [1]. Currently, Chicago classification using high-resolution manometry (HRM) as the diagnostic criteria of achalasia has become an international standard [2]. Consequently, the morphologic classification based on traditional Barium esophagogram has become less clinically important.

**Fig. 1:**
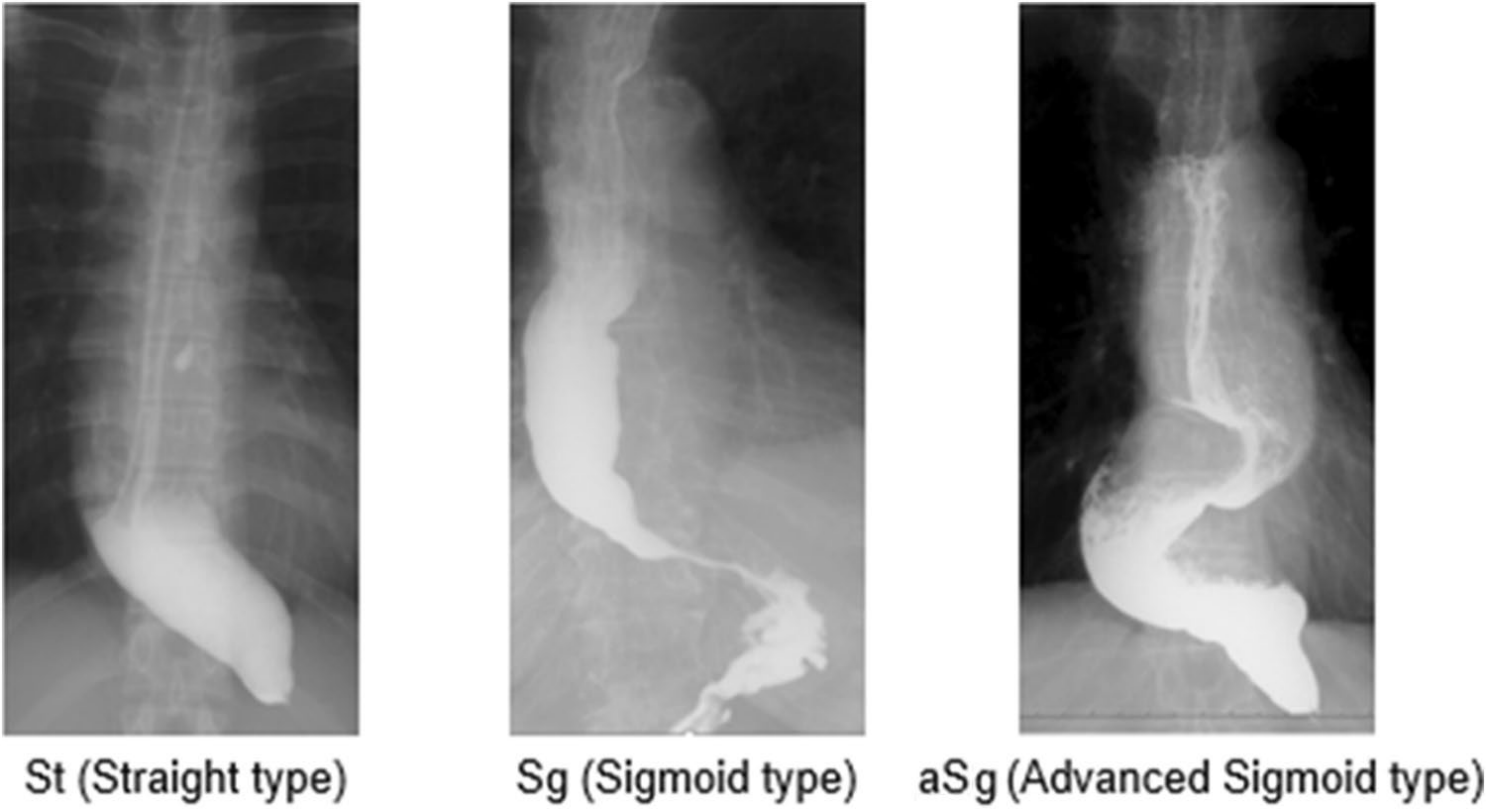
New Japanese X-ray classification system for esophageal achalasia was revised in 2012. Esophageal achalasia is classified into 3 types, St (Straight type), Sg (Sigmoid type) and aSg (Advanced Sigmoid type), based on the X-ray findings. If there is a bending of the esophagus, draw a straight line to the esophagus long axis direction. Determine the angle *α* at the intersection of two straight lines. (St: *α* ≥ 135°, Sg: 90° ≤ *α* ≤ 135°, aSg: *α* < 90°)

Recently, the Japanese classification system for esophageal achalasia was revised after a long interval of 30 years [3]. In this system, achalasia is classified into three types: St (straight type), Sg (sigmoid type), and aSg (advanced sigmoid type), based on its X-ray findings (Fig. 1). In comparison with its previous version issued in 1983, this new system aims at more practical classification based on clinical pathology. However, the system has been limitedly used in Japan and has not been fully validated in terms of its demographical significance and predictive capability of postoperative outcomes.

The purpose of this study was to clarify the validity of new Japanese classification system for esophageal achalasia, as an index of patient characteristics and as a predictor of operative and mid/long-term postoperative outcomes.

## Patients and methods

The consecutive 59 patients with a definitive diagnosis of esophageal achalasia who underwent laparoscopic Heller–Dor surgery (LHD) by a single operative team from April 2005 to April 2018 were enrolled in the study. A signed informed consent was obtained from all patients prior to surgery. Diagnosis of achalasia was confirmed by traditional esophageal manometry. The cases were retrospectively classified into three disease types according to new Japanese classification system. We enrolled 34 St and 25 Sg cases. Since patients with aSg disease were limited (*n* = 4), together Sg and aSg to one group (Sg/aSg) were compared with St group based on morphological classification. The following data were collected and compared between above two groups: (1) patients’ characteristics: age, gender, body mass index (BMI), preoperative duration of symptoms, preoperative treatment (i.e., balloon dilatation and Ca inhibition), maximum transverse diameter of the esophagus at Barium esophagography, co-morbidity; (2) operative findings: operation time, blood loss, intraoperative complications; (3) postoperative course: postoperative complications, clinical symptoms (such as residual passage disturbance and chest pain). Postoperative subjective symptoms were assessed within 1 year after surgery. Treatment and symptoms were examined from those extracted from medical charts. We evaluated postoperative passage disturbance using the following three parameters: dysphagia, resistance at endoscopy passage at esophagogastric (EG) junction, and body weight change. We evaluated dysphagia using 3 scales: (i) persistent, (ii) intermittently and (iii) no. “Endoscopy passage” was classified into 2 grades: (i) with resistance and (ii) without resistance. “Body weight change” was classified into 3 grades: (i) loss, (ii) no, and (iii) gain in within 1 year after surgery compared with preoperative. (4) Esophageal motor function tests (i.e., preoperative and postoperative manometric values of LES pressure). Esophageal manometry with computerized 3-D pressure imaging was performed in 31 cases with a stepwise manual pullback method using a manometric assembly with 8 radial side holes (Adult Anorectal Sidehole Catheter A-E1-ASH-1, Dentsleeve International, Ltd., Mississauga, Ont, Canada). The 8 channels were perfused with degassed, distilled water at a rate of 0.3 ml/min using a low-compliance pneumohydraulic pump. Postoperative esophageal manometry was generally performed between 6 months and 1 year after surgery; (5) Perioperative 24-h pH monitoring test was used to evaluate the reflux (SLEUTH ZepHr: SANDHILL SCIENTIFIC, USA). This study was reviewed and approved by the institutional review board of Osaka University (No. 08226-6).

## Surgical procedure

**Fig. 2:**
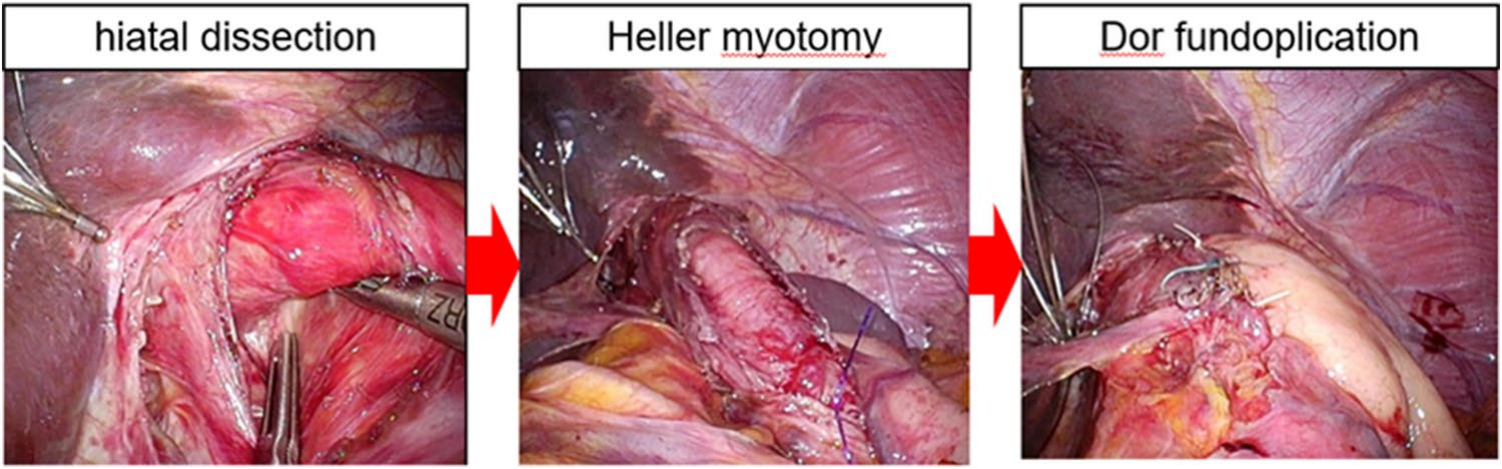
Surgical technique of the hiatal dissection, Heller myotomy, and Dor fundoplication

Our surgical procedure for LHD is previously described in detail elsewhere [4–7]. Briefly, an esophagomyotomy of 5 cm long is performed after securing the abdominal esophagus. This myotomy is extended 2 cm onto the gastric side. During myotomy, a CRE balloon (C.R.E.; Boston Scientific, Natick, Mass., USA) is placed at the esophagogastric junction, with the aid of guide wire. The CRE balloon is gently inflated with water and deflated after cutting the circular muscle fibers. A 180° Dor anterior fundoplication is then fashioned and the most cranial sides are fixed to the diaphragmatic fascia to reduce tension and to avoid axial twisting of the distal esophagus. We performed a similar procedure regardless of the type of achalasia (Fig. 2).

### Statistical analysis

The data from the St cases and from the Sg cases were analyzed statistically using the computer program JMP8.0.2 (SAS Institute, Cary, NC, USA). In all items, the comparison between the two groups was made at the median value. In tables, they were expressed as median [25th percentile, 75th percentile]. The Wilcoxon-Mann–Whitney test and Fisher’s exact test were used for statistical analysis, and *p *< 0.05 was considered to be statistically significant.

## Results

### Patient characteristic

Thirty-four patients in the St group and 25 in the Sg group were enrolled, respectively (Table 1). The Sg group was consisted of 21 Sg patients and 4 aSg patients. The age of St group was significantly younger than Sg group [median (25th percentile, 75th percentile); 38 (26, 46) years vs. 56 (39, 68) years, *p* < 0.01]. No significant difference was observed in gender and BMI between the two groups. Preoperative duration of disease of St group was shorter than that of Sg [39 (24, 64) months vs. 120 (48, 240) months, *p* < 0.01]. Body weight loss in 1 year of St group was greater than that of Sg (5 kg vs. 0 kg, *p *= 0.02). The frequency of using preoperative balloon dilatation and Ca inhibitor was not significantly different. There was no significant difference in maximum transverse diameter of the esophagus between the two groups.

**Table 1:** Patients’ characteristics

Variables	St (*N* = 34)	Sg (*N* = 25)	*p* value
Age (years)	38 [26, 46]	56 [39, 68]	<0.01
Gender (M/F)	20/14	10/15	0.15
BMI (kg/m^2^)	19 [18, 21]	21 [18, 24]	0.10
Preoperative duration of disease (months)	39 [24, 64]	120 [48, 240]	<0.01
Body weight loss (kg)	5 [0, 8]	0 [0, 2]	0.02
Preoperative treatment (*n*)			
Balloon dilatation	3	7	0.05
Ca inhibitor	8	7	0.69
Maximum transverse diameter at the esophagography (mm)	42 [36, 49]	44 [39, 50]	0.63

*NS* not significant

### Intraoperative findings

All the procedures were completed under laparoscopy and open conversion was not required for either group (Table 2). There was no significant difference in operation time [median (25th percentile, 75th percentile); 217 (188, 248) min vs. 205 (189, 240) min]. The blood loss was negligible and did not exceed 100 mL in most patients of the two groups. The incident of intraoperative mucosal perforation was only one case in both groups.

**Table 2:** Intraoperative findings

Variables	St (*N* = 34)	Sg (*N* = 25)	*p* value
Open conversion (*n*)	0	0	N/A
Operative time (min)	217 [188, 248]	205 [189, 240]	0.32
Blood loss (ml)	13 [10, 30]	10 [10, 25]	0.96
Intra operative complications			
Mucosal injury [*n* (%)]	1 (3)	1 (4)	0.79

*NS* not significant

### Esophageal manometric profiles

All preoperative and postoperative esophageal manometric profiles were reviewed (Table 3). The manometry was performed in 21 patients (62%) of St group and in 8 (32%) of Sg group, respectively. The evaluation items were the perioperative average pressure and the decreasing rate before and after the operation. A significant reduction was observed in the pressure resistance of LES in all patients, and no significant differences were identified in any of manometric values between the two groups.

**Table 3:** Perioperative esophageal manometry

Variables	St (*N* = 21)	Sg (*N* = 8)	*p* value
Mean pressure (mmHg)			
Preoperative	28 [22, 33]	18 [14, 28]	0.16
Postoperative	10 [8, 13]	11 [8, 14]	0.86
Decreasing rate (%)	52 [84, 34]	43 [54, 31]	0.18

*NS* not significant

### 24-h pH monitoring

Postoperative 24-h pH monitoring test was performed in 22 patients (65%) of St group and in 15 patients (60%) of Sg group, respectively (Table 4). A postoperative DeMeester score above 14.7 was observed in 9 (43%) patients in St group and in 6 (40%) patients in Sg group, respectively. There were no statistically significant differences in postoperative values of 24-h pH monitoring in either of the two groups. All evaluation items were examined by average value.

**Table 4:** Postoperative 24-h pH monitoring

Variables	St (*N* = 22)	Sg (*N* = 15)	*p* value
Fraction time pH < 4.0 (%)	2 [0, 13]	1 [0, 3]	0.62
Number of reflux (*n*)	14 [3, 187]	16 [3, 52]	0.65
Number of long reflux (> 5 min) (*n*)	1 [0, 6]	0 [0, 1]	0.18
Longest time of reflux (min)	4 [0, 32]	3 [1, 11]	1.00
DeMeester score > 14.7 [*n* (%)]	9 (43)	6 (40)	0.86

*NS* not significant

### Postoperative course

Table 5a depicts postoperative treatment and symptoms of the two groups. No differences were observed in postoperative symptom and treatment between the two groups.

**Table 5:** Postoperative course

Variables	St (*N* = 34)	Sg (*N* = 25)	*p* value
(a)			
Complication [*n* (%)]	0 (0)	0 (0)	N/A
Postoperative symptom [*n* (%)]			
Heart burn	2 (6)	3 (12)	0.29
Chest pain	13 (39)	3 (12)	0.06
Postoperative treatment [*n* (%)]			
Pneumatic dilatation	3 (9)	1 (4)	0.47
Calcium inhibitor	9 (26)	4 (16)	0.35
Antacid agents	8 (24)	5 (20)	0.76

Dysphagia (*n* = 53)	Endoscopy passage (*n* = 48)	Body weight change (*n* = 52)
(b)		
Persistent	With resistance	Loss
2: St (2), Sg (0)	2: St (0), Sg (2)	2: St (1), Sg(1)
Intermittently	Without resistance	No
29: St (16), Sg (13)	48: St (27), Sg (19)	9: St (4), Sg(5)
No		Gain
24: St (13), Sg (11)		43: St (26), Sg(17)

*NS* not significant

There were 31 patients (58%) who had persistent and intermittently dysphagia, but there was no significant difference between these two groups. There were two cases with resistance of endoscopy passage and both cases were classified into Sg type. Body weight loss was only seen in 2 cases, 1 in St and the other in Sg, respectively (Table 5b).

## Discussion

We have unique Japanese system besides Chicago system for the diagnosis and classification of esophageal achalasia. In 2012, this classification system was revised after an interval of 30 years. In this revised system, achalasia is classified into three types: St (straight type), Sg (sigmoid type), and aSg (advanced sigmoid type), based on its X-ray findings. However, there is no detailed report that evaluates its clinical significance as an index of patient characteristics and as a predictor of operative and mid/long-term postoperative outcomes. To our knowledge, this study is one of the latest and largest validation reports in surgical literature.

Our study first demonstrated that age of St patients is lower than that of Sg, and preoperative duration of disease is longer in Sg group than that in St group. In 1987, Hirashima reported that there might be an association between disease type of previous Japanese classification and duration of disease [8]. In his report, he speculated that straight type disease might progress into sigmoid type disease after long duration of morbidity. Our data also support this hypothesis, since our Sg patients had longer preoperative morbidity period and subsequently older at surgery.

We also obtained the same result; Japanese classification system may indicate the progress of the disease types. The association between the age and disease type reflects the preoperative duration of disease.

However, the treatment outcomes showed no significant difference between the two groups. Intraoperative findings showed no difference in disease types. This indicates that it is not related to the degree of difficulty of the surgery and the disease type. Also, we initially hypothesized that St group had better outcomes than Sg group with postoperative symptoms and treatment. However, there was no difference between the disease types, because postoperative symptoms and treatment were evaluated only one year after surgery. It is necessary to examine more long-term outcomes. In addition, 59 cases are few to morphologically study of esophagus achalasia. We need to accumulate more cases and re-examine them. For objective evaluation of reflux after laparoscopic Heller–Dor, we performed a 24-h pH monitoring test, but there was no difference between the two groups. From this result, it was considered that the esophagus X-ray classification does not affect postoperative gastroesophageal reflux disease.

Postoperative passage disturbance with different severity was seen in more than half of the cases. Our study first demonstrated that these “remnant” symptoms were equally seen in any types of achalasia. This was considered mainly due to persistence of dismotility in the esophageal body, which is not repaired with traditional myotomy [9]. As for endoscopy passage at EG junction, again we could not show its difference among different disease types. This might reflect the appropriateness of myotomy, though still not conclusive due to the very small number of cases (only 2 cases with difficulty in insertion). Likewise, no difference was seen in postoperative weight loss between St and Sg types (only 1 case in St and 1 in Sg). These data strongly indicate that the total number of patients involved was not sufficient to discuss any differences in postoperative outcomes among different disease types. Further accumulation of clinical cases, ideally in multicenter setting, is considered mandatory to fully validate new Japanese classification system for esophageal achalasia.

There are two limitations in this study: (1) Sample size was too small. As a result, each group became too small to be compared with one another. The three-group comparison, i.e., St vs. Sg vs. aSg, therefore, became impossible. (2) Patients’ clinical symptoms were not adequately evaluated objectively. Symptoms data were obtained based on “narrative” description in the medical charts. We should evaluate symptoms using the Eckardt score. Also, we should do the TBE (Timed barium esophagogram) as the evaluation of esophageal clearance.

In the future, we will further accumulate achalasia cases. Considering international Chicago classification and this new Japanese classification system will lead to the elucidation of the clinical pathology of achalasia.

## Conclusions

The new Japanese classification system may give additional insight and information in understanding epidemiology of esophageal achalasia; however, our study failed to demonstrate “inter-disease type” differences in surgical outcomes and prognoses. Further accumulation of clinical cases is definitely necessary to clarify its predictive capability.

## Abbreviations

St: Straight type
Sg: Sigmoid type
aSg: Advanced Sigmoid type
